# Editorial Note: Effect of intrinsic foot muscles training on foot function and dynamic postural balance: A systematic review and meta-analysis

**DOI:** 10.1371/journal.pone.0330111

**Published:** 2025-08-12

**Authors:** The PLOS One Editors

After this article [1] was published, it was brought to the attention of the *PLOS One* Editors that there is a closely-related systematic review that was previously registered in PROSPERO and published as part of a thesis [2,3]; this study was later published in [4].

A member of the *PLOS One* Editorial Board advised that despite partial overlap in the theme and included studies, the two articles [1] and [4], differ in their framing, analytical direction, and conclusions.

The *PLOS One* Editors issue this Editorial Note to make readers aware of the related work [2,3] that was publicly available at the time of publication but was not cited.
